# Beyond leaf mass per area: anatomical and economic drivers of toughness in evergreen and deciduous oaks

**DOI:** 10.1111/tpj.71217

**Published:** 2026-11-11

**Authors:** Rubén Martín‐Sánchez, Domingo Sancho‐Knapik, José Javier Peguero‐Pina, Juan Pedro Ferrio, Yusuke Onoda, Eustaquio Gil‐Pelegrín

**Affiliations:** ^1^ Departamento de Sistemas Agrícolas, Forestales y Medio Ambiente Centro de Investigación y Tecnología Agroalimentaria de Aragón (CITA) Zaragoza Spain; ^2^ Instituto Agroalimentario de Aragón ‐IA2‐ (CITA‐Universidad de Zaragoza) Zaragoza Spain; ^3^ Unidad de Suelos y Riegos Centro de Investigación y Tecnología Agroalimentaria de Aragón (CITA), Estación Experimental Aula Dei (EEAD‐CSIC) Zaragoza Spain; ^4^ Estación Experimental de Aula Dei, Consejo Superior de Investigaciones Científicas (EEAD‐CSIC) Zaragoza Spain; ^5^ Graduate School of Agriculture Kyoto University Kyoto 606‐8502 Japan

**Keywords:** leaf habit, *Quercus*, leaf mass per area, leaf mechanical resistance, cell wall composition, construction cost

## Abstract

We examined how leaf habit (deciduous versus evergreen) shapes the structural, mechanical, and chemical traits of oaks (genus *Quercus*), aiming to determine whether evergreen leaf toughness arises from intrinsic chemical investment or greater tissue allocation per unit area. Leaves from 33 oak species were sampled, focusing on interveinal lamina. Morphological traits (LMA, thickness, density), mechanical properties, and cell wall composition (cellulose, hemicellulose, lignin, cutin, nitrogen) were measured. The fiber fraction composition was used to estimate the construction costs and heat of combustion. Comparisons between deciduous and evergreen species were made on area‐ and mass‐bases. Evergreen leaves had higher leaf mass per area, thickness, and toughness than deciduous leaves, with partial overlap in LMA. Area‐based investments in cell wall polymers and construction costs were consistently higher in evergreens. Mass‐based fiber construction costs were lower in evergreens, reflecting the dominance of metabolically inexpensive polysaccharides, whereas deciduous fibers contained more energetically costly compounds such as lignin and cutin. Evergreen leaf toughness is primarily driven by tissue quantity (LMA) and specific anatomical reinforcement rather than intrinsic fiber cost. Deciduous leaves are mechanically weaker, despite higher mass‐based fiber cost. These results highlight a decoupling between chemical investment and mechanical properties and show how leaf structural design underpins functional divergence between leaf habits.

## Significance Statement

Evergreen oak leaves achieve greater mechanical strength not because they build intrinsically more expensive structural tissues, but because they invest more heavily in relatively inexpensive structural carbohydrates per unit leaf area. By linking leaf mechanics, fiber chemistry, and construction costs within the same interveinal tissue, this study provides a mechanistic understanding of the structural basis of sclerophylly and leaf persistence.

## INTRODUCTION

Plant leaf habit, whether deciduous or evergreen, reflects evolutionary responses to environmental pressures, particularly those related to resource availability or climate (Reich et al., 2007; Sancho‐Knapik et al., 2021). In resource‐poor or seasonally stressful environments, evergreen species are often associated with conservative resource‐use strategies, maintaining photosynthetically active tissues over multiple growing seasons despite lower photosynthetic rates per unit time (Chabot & Hicks, 1982; Wright et al., 2004). This strategy allows them to compensate for the higher “costs” of leaf construction over a longer lifespan, that is, the energetic and biochemical resources required to synthesize leaf tissues. According to the leaf economics spectrum (LES), evergreen leaves are generally characterized by high construction costs, low nitrogen concentration, and extended longevity. In contrast, deciduous species, particularly inhabiting more mesic or fertile environments, tend to invest in short‐lived but more productive foliage, maximizing rapid return on investment (Reich et al., 1999; Wright et al., 2004).

One of the main features more associated with evergreen than with deciduous leaves is sclerophylly, commonly quantified through the leaf mass per area ratio (LMA) (Groom & Lamont, 1999). Although sclerophylly is often treated as a qualitative distinction between “hard” and “soft” leaves, it can also be viewed as a continuous trait reflecting variation in LMA. Following Flexas et al. (2014), species with LMA > 120 g m^−2^ can be classified as sclerophylls, although the threshold varies among authors (cf. Lamont & Lamont, 2025). Species below this threshold are classified here as orthophylls (Fosberg, 1961; Cowling & Campbell, 1983). Similarly, leaf habit represents a categorical classification of leaf lifespan (LLS), with species having LLS > 12 months considered evergreen and those with LLS < 12 months considered deciduous. Evergreen leaves typically exhibit higher LMA values than deciduous leaves, reflecting greater structural reinforcement and chemical investment (Poorter et al., 2006). LMA itself is composed of two main components: leaf thickness (LT) and leaf density (Witkowski & Lamont, 1991), which reflect underlying anatomical traits, such as mesophyll packing, cell wall thickness, cuticle thickness and fiber content, that may be different between species with evergreen and deciduous leaves (Alonso‐Forn et al., 2023; Sancho‐Knapik et al., 2021). LMA also correlates with various ecological and functional leaf traits as a widely used proxy for leaf toughness, that is, leaf resistance to crack propagation (Niinemets, 2001; Reich et al., 1999). However, LMA does not always accurately reflect leaf mechanical resistance (Alonso‐Forn et al., 2023). Recent work has emphasized the need to measure leaf mechanical properties more directly through traits such as strength, work‐to‐shear, and maximum force (*F*_max_). *F*_max_ reflects the maximum load required to penetrate or fracture the leaf lamina and therefore provides a direct measure of leaf resistance to mechanical damage (Kitajima et al., 2012; Onoda et al., 2011; Sancho‐Knapik et al., 2023). These traits can influence not only survival under abiotic stress, but also resistance to herbivory, pathogen attack, and wind damage (Hanley et al., 2007).

A crucial, yet often overlooked, aspect of leaf mechanical properties is the chemical composition of the cell walls. The construction of hard leaves (sclerophyllous species) involves not only anatomical investment but also metabolic investment in cell wall biopolymers such as cellulose, hemicellulose, lignin, cutin, and structural proteins (Alonso‐Forn et al., 2023). Each of these components entails different biosynthetic costs; for instance, lignin and cutin are metabolically “expensive” to synthesize—meaning that their production requires greater energetic investment—while cellulose and hemicellulose are comparatively cheaper to produce (Poorter & Villar, 1997; Villar & Merino, 2001). Therefore, variations in the proportion of these compounds will influence both leaf construction costs and mechanical properties. In mechanical terms, leaf laminas are complex structures in which cellulose, hemicellulose, lignin, cutin, and structural proteins contribute differently to cell wall architecture and function. For example, lignin contributes to structural reinforcement and resistance to tearing and bending, whereas cellulose‐rich cell walls have been associated with enhanced tensile strength and mechanical support (Kitajima et al., 2012; Sobrado, 2009). Consequently, differences in cell wall composition may have important consequences for leaf mechanical properties beyond their effects on construction costs. It has been traditionally assumed that the higher construction cost of evergreen or hard leaves is due to an investment in costly compounds such as lignin and cutin (Miller & Stoner, 1979; Osunkoya et al., 2008; Sobrado, 2009). This assumption was largely based on the high biosynthetic costs and structural roles attributed to these compounds. However, recent evidence indicates that cellulose content may play a more significant role in determining leaf strength than previously thought, challenging earlier assumptions about construction cost patterns (Alonso‐Forn et al., 2023; Escudero et al., 2017; Kitajima et al., 2012). Here, we use the term fiber fraction to refer to the detergent‐insoluble structural fraction of the leaf, comprising mainly cell wall biopolymers such as cellulose, hemicellulose, lignin and cutin, rather than to specific anatomical tissues such as sclerenchyma.

Few studies have systematically quantified cell wall composition and its investment cost in relation to LMA and mechanical properties across species with contrasting leaf habits (Escudero et al., 2017; Villar & Merino, 2001). Previous work has established that evergreen oaks exhibit higher LMA, greater mechanical strength, and a larger investment in structural tissues than deciduous species (Alonso‐Forn et al., 2023; Kitajima et al., 2012; Sancho‐Knapik et al., 2021). However, several issues remain unresolved. Most studies have focused either on the anatomical basis of leaf toughness or on cell wall chemistry but rarely integrate both perspectives with construction costs at the interveinal level, where mechanical properties are actually measured. Furthermore, lignin and cutin—two chemically and functionally distinct, yet costly, polymers—are commonly quantified together within the fiber fraction (Alonso‐Forn et al., 2023; Sancho‐Knapik et al., 2021; Van Soest, 1963), and the relative abundance of fiber components is generally expressed on a dry mass basis, overlooking differences in LMA and leaf internal structure. Consequently, the independent contribution of individual wall components, particularly cutin and lignin, to leaf mechanical properties and construction costs remains poorly understood. Moreover, many previous studies have sampled phylogenetically diverse species, making it difficult to disentangle evolutionary lineage effects from trait‐based adaptations or to determine whether overlapping LMA values between leaf habits translate into similar mechanical properties.

Because leaf mechanical resistance is measured in the interveinal lamina, understanding its structural basis requires that anatomical, chemical, and energetic traits be quantified within the same tissue. The present study focuses on a diverse set of deciduous and evergreen oak species (genus *Quercus* L.). Oaks provide an ideal taxon to examine leaf functional variation while minimizing confounding phylogenetic effects. Moreover, genus *Quercus* encompasses species with a wide range of LMA values, mechanical properties, and cell wall compositions, and has been extensively used in trait‐based ecological research (Gil‐Pelegrín et al., 2017). We hypothesized that the greater mechanical resistance of evergreen than deciduous leaves arises primarily from differences in the amount and composition of the interveinal structural fraction and that this is done without additional construction costs. To test this hypothesis, we addressed three objectives: (1) to characterize leaf morphological and mechanical traits between deciduous and evergreen oaks and the relationships among these traits; (2) to quantify the chemical composition of the interveinal fiber fraction distinguishing lignin from cutin and expressing all components on both a leaf area and dry mass basis; and (3) to estimate the construction cost of this interveinal fiber fraction to determine whether differences between leaf habits reflect the production of more expensive tissues or simply a greater allocation to the same structural compounds per unit leaf area. By integrating anatomical, chemical, mechanical, and energetic perspectives within the same leaf tissue, this study aimed to provide a mechanistic understanding of the structural strategies underlying leaf habit in oaks.

## RESULTS

### Trait differences between deciduous and evergreen species

A summary of the main results is given in Table 1. Both leaf mass per area traits, leaf mass per area of the whole leaf (LMA_l_) and interveinal leaf mass per area (LMA_i_) are strongly correlated (*R* ^2^ = 0.916, *P* < 0.001). LMA_i_ is on average 14.9% lower than LMA across all species. Within the oak species studied, evergreens (EVE) exhibit on average a significantly higher LMA_i_ than deciduous (DEC) species (EVE = 122 ± 28.4 g m^−2^; DEC = 72.2 ± 24.6 g m^−2^; *P* < 0.001) (Figure 1a, Table 1). They also have significantly thicker leaves than deciduous species (Figure 1b, Table 1). However, leaf density was significantly higher in deciduous than in evergreen species (Figure 1c, Table 1). Evergreen species also show a significantly greater maximum force (*F*_max_) compared with deciduous species (Figure 1d, Table 1).

**Table 1:** Mean values (± standard deviation) of morphological, chemical, and construction cost traits for the 33 *Quercus* species included in this study (20 deciduous, DEC; and 13 evergreen, EVE; species)

Trait	DEC	EVE	Significance
LA	23.905 ± 14.182	10.925 ± 8.470	**
LMA_l_	82.964 ± 28.015	146.056 ± 34.135	***
LMA_i_	72.186 ± 24.633	121.725 ± 28.388	***
LT	0.137 ± 0.050	0.261 ± 0.057	***
Density	0.531 ± 0.063	0.466 ± 0.045	**
*F*_max_	1.394 ± 0.760	6.159 ± 1.694	***
S	0.444 ± 0.242	1.960 ± 0.539	***
SS	3.148 ± 0.927	7.481 ± 1.567	***
WF	65.082 ± 40.450	329.926 ± 180.834	***
SWF	0.458 ± 0.158	1.223 ± 0.504	***
Cell content	69.970 ± 4.574	63.824 ± 2.778	***
Fiber fraction	30.030 ± 4.574	36.176 ± 2.778	***
Fib_m^2^	24.343 ± 7.254	53.024 ± 14.298	***
Cel_m^2^	7.840 ± 2.588	18.954 ± 5.311	***
Hem_m^2^	7.703 ± 2.314	15.073 ± 4.352	***
Lig_m^2^	1.867 ± 0.567	3.587 ± 1.419	***
Cut_m^2^	3.518 ± 1.439	5.953 ± 1.414	***
N_m^2^	1.210 ± 0.449	1.448 ± 0.452	*
Hc_m^2^	430.031 ± 133.938	851.939 ± 214.701	***
CC_m^2^	31.912 ± 10.006	61.903 ± 15.916	***
Cel_fib	36.870 ± 3.388	42.758 ± 2.270	***
Hem_fib	36.451 ± 2.056	34.007 ± 2.367	**
Lig_fib	8.940 ± 1.730	8.195 ± 2.643	ns
Cut_fib	16.310 ± 3.409	13.711 ± 2.567	*
N_fib	1.517 ± 0.539	1.269 ± 0.385	ns
N_Total	1.71 ± 0.35	1.19 ± 0.23	***
Hc_fib	19.263 ± 0.479	18.876 ± 0.286	**
CC_fib	1.47 ± 0.05	1.44 ± 0.03	**

Morphological and mechanical variables are based on species means calculated from eight leaves sampled from five individuals per species. Chemical variables were obtained from one composite interveinal sample per species. The level of significance resulting from statistical tests comparing evergreen and deciduous species is indicated by asterisks.

* *P* < 0.05.

** *P* < 0.01.

*** *P* < 0.001.

ns, not significant.

**Figure 1:**
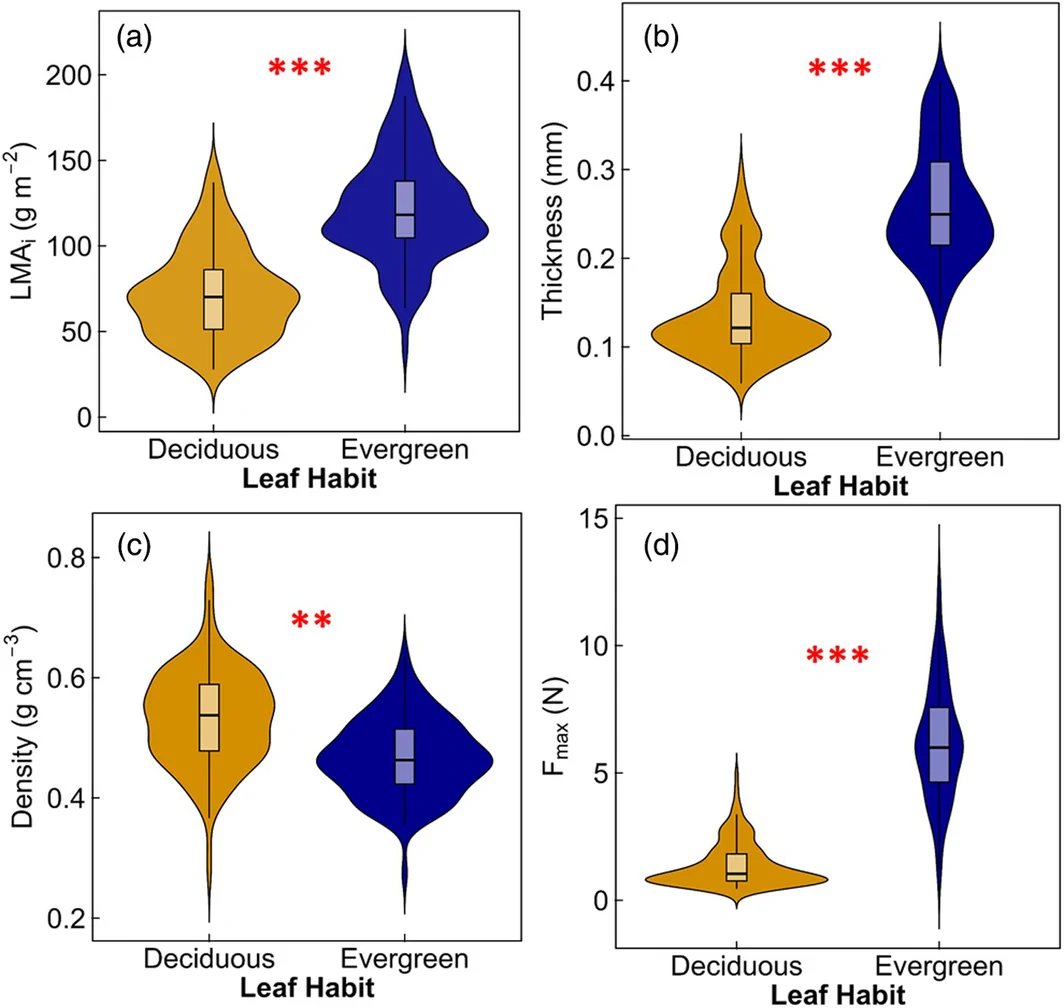
Violin plots for the four single‐value traits: (a) interveinal leaf mass per area (LMA_i_), (b) leaf thickness, (c) leaf density, and (d) maximum force (*F*_max_). Each panel shows trait distributions for deciduous (orange, *n* = 20) and evergreen (blue, *n* = 13) species. Eight leaves were sampled from five individuals per species. The inner boxplots show the median (horizontal line), the first and third quartiles (lower and upper boundaries of the box), and whiskers extending to the most extreme values within 1.5 × the interquartile range; outliers are not shown. Red asterisks indicate the significance level of differences between leaf habits: *P* < 0.05 (*), *P* < 0.01 (**), *P* < 0.001 (***).

The ANOVA detected significant overall differences among the four subgroups defined for each trait—deciduous species below versus above their median, and evergreen species below versus above their median (see Materials and Methods for further details)—for all variables (LMA_i_, LT, density, and *F*_max_; *P* < 0.05). Subsequent Tukey's HSD tests indicated that, for LMA_i_, evergreen species below the evergreen median did not differ significantly from deciduous species above the deciduous median. Likewise, for leaf density, evergreen species above the evergreen median did not differ significantly from deciduous species below the deciduous median. In contrast, all pairwise comparisons between corresponding deciduous and evergreen subgroups remained significantly different for LT and *F*_max_. Although the thickest deciduous species exhibited, on average, thicker leaves than the thinnest evergreen species, these subgroups remained statistically distinct. Similarly, evergreen species with the lowest *F*_max_ values still exhibited significantly higher *F*_max_ than deciduous species with the highest values. Kernel density distributions for LMA_i_, leaf thickness (LT), density, and *F*_max_ are shown in Figure S4. The overlap coefficient (OVL) indicated the greatest overlap between leaf habits for density (OVL = 0.567), followed by LMA_i_ (OVL = 0.363) and then, leaf thickness (OVL = 0.237), whereas *F*_max_ showed only minimal overlap (OVL = 0.094).

The LMA_i_ is strongly correlated with *F*_max_ (Figure 2a). Thickness also shows a highly significant correlation both with LMA_i_ (*R* ^2^ = 0.920, *P* < 0.001; data not shown) and with *F*_max_ (Figure 2b). Even after removing the effect of thickness (i.e., using specific strength, SS), a significant correlation with LMA_i_ remained (*R* ^2^ = 0.448, *P* < 0.001) (Figure S5). For *F*_max_ versus LMA_i_, the interaction was significant (*F*_1,29_ = 5.10, *P* = 0.032), indicating different slopes between DEC and EVE, so separate regression lines are shown in Figure 2a. In contrast, for *F*_max_ versus LT (*P* = 0.266; Figure 2b) and specific strength (SS) versus LMA_i_ (*P* = 0.788; Figure S5), the interactions were not significant, indicating similar slopes between groups, and a single global regression line is shown (Figure 2b, Figure S5).

**Figure 2:**
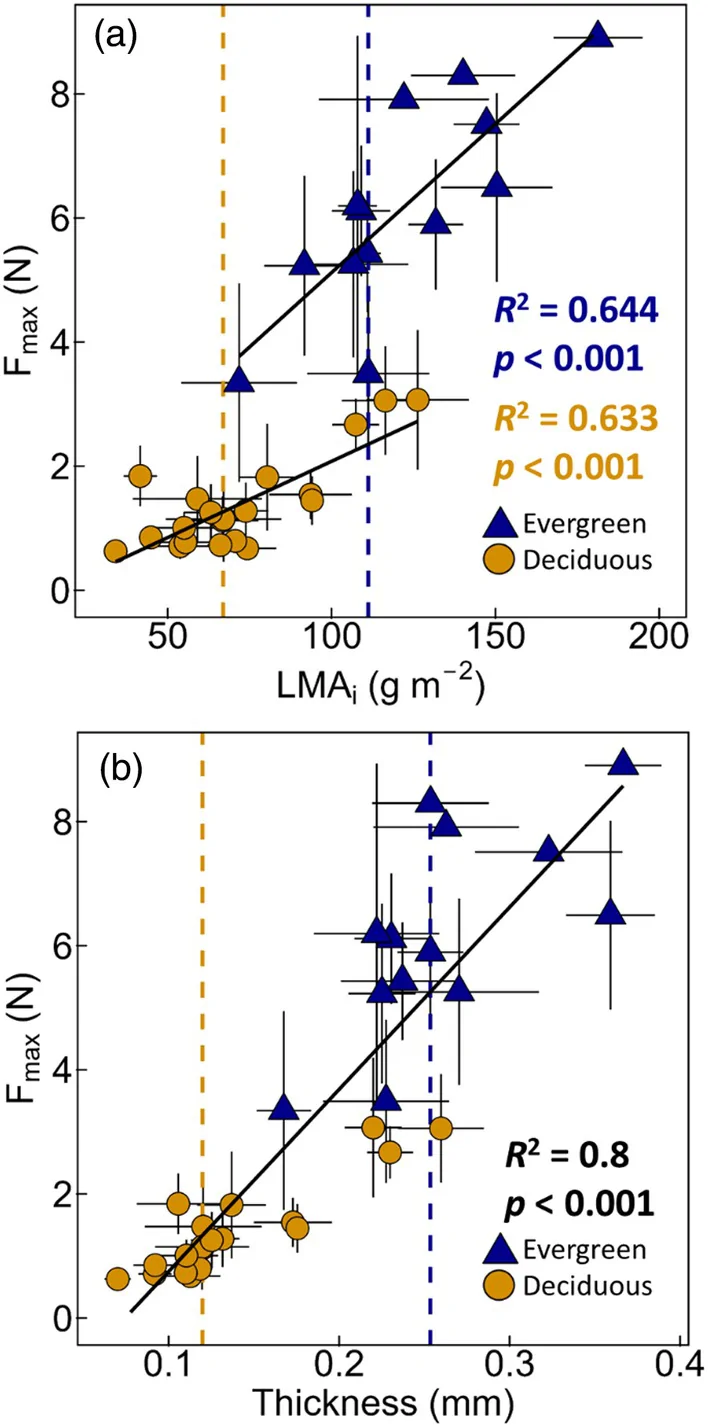
Correlation between interveinal leaf mass per area (LMA_i_) and maximum force (*F*_max_) (a), and between leaf thickness and maximum force (b). Each point represents the mean ± SD of eight leaves sampled from five individuals for one species. Blue triangles represent evergreen species (*n* = 13) and orange circles deciduous ones (*n* = 20). Dashed colored lines mark the median of each leaf habit for the *x*‐variables. Linear regression lines were fitted to the data. In panel (a), the interaction between leaf habit and LMA_i_ was significant (*P* = 0.032), so separate regression lines are shown for deciduous and evergreen species (both regressions: *P* < 0.001). In panel (b), the interaction between leaf habit and thickness was not significant (*P* = 0.266), so a single regression line was fitted across species (*P* < 0.001).

After the neutral detergent fiber (NDF) and alcohol insoluble residue (AIR) extractions (see Materials and Methods for details), evergreen species show, as a percentage of the dry mass, significantly more fiber fraction and fewer cell content than deciduous species (Table 1). The combustion of each polymer is: 16.03 ± 0.11 kJ g^−1^ for xylan (as a representative of hemicellulose), 16.80 ± 0.67 kJ g^−1^ for cellulose, 21.91 ± 0.33 kJ g^−1^ for lignin, 22.62 ± 0.31 kJ g^−1^ for gluten (as a representative of plant proteins), and 30.21 ± 0.18 kJ g^−1^ for cutin (data not shown; see Materials and Methods for further details on the calculation of the combustion energy of the different cell wall components).

### Composition on an area basis

When the fiber content is expressed per square meter via the LMA_i_, the difference between both leaf habits becomes more pronounced (Table 1). If the quantity of the different cell wall components per square meter is compared within both leaf habits, evergreen species also show a significantly higher investment in all four major cell wall components per square meter (*P* < 0.001 for each) (Figure 3a, Table 1). Total nitrogen content per square meter is significantly higher in evergreen species compared with deciduous ones (Figure 3b, Table 1). Evergreens also exhibit significantly greater heat of combustion than deciduous species (Figure 3c, Table 1). Regarding construction cost, evergreens require a significantly higher investment to construct a square meter of leaf tissue than deciduous species (Figure 3d, Table 1).

**Figure 3:**
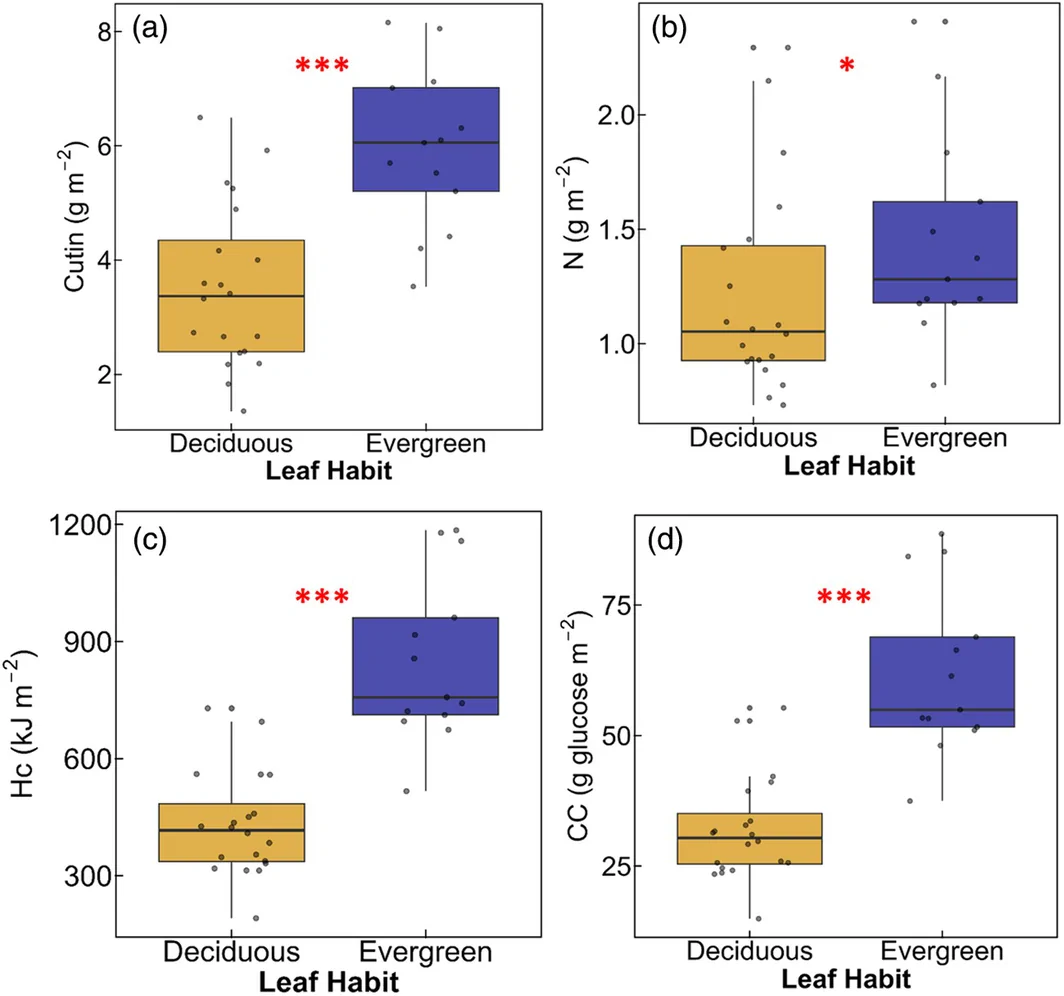
Boxplots of traits on leaf area basis comparing deciduous (orange, *n* = 20) and evergreen (blue, *n* = 13) species: (a) Cutin per square meter, (b) Total nitrogen content per square meter, (c) Heat of combustion per square meter, and (d) Construction cost per square meter. Chemical composition was determined from one composite interveinal sample per species, analyzed in triplicate (technical replicates), and the mean value was used in all statistical analyses. Each dot represents the mean value for an individual species. The boxplots show the median (horizontal line), the first and third quartiles (lower and upper boundaries of the box), and whiskers extending to the most extreme values within 1.5× the interquartile range; outliers are not shown. Red asterisks indicate statistical significance levels based on *t*‐test/Wilcoxon tests: *P* < 0.05 (*), *P* < 0.01 (**), *P* < 0.001 (***).

All the cell wall components correlated significantly with LMA_i_ (Figure 4). *Post hoc* comparisons revealed differences in scaling intensity among components, with cellulose (slope = 0.175 ± 0.012) and hemicellulose (slope = 0.128 ± 0.009) showing relatively steeper responses than cutin (slope = 0.044 ± 0.005) and lignin (slope = 0.027 ± 0.004). This pattern was formally supported by a significant interaction between LMA_i_ and component identity (*F* = 72.59, *P* < 0.001), confirming that scaling relationships differ among compounds. Additionally, cutin content per unit leaf area was positively correlated with specific punch strength (SS; *R* ^2^ = 0.362, *P* < 0.001), a thickness‐independent measure of leaf mechanical resistance.

**Figure 4:**
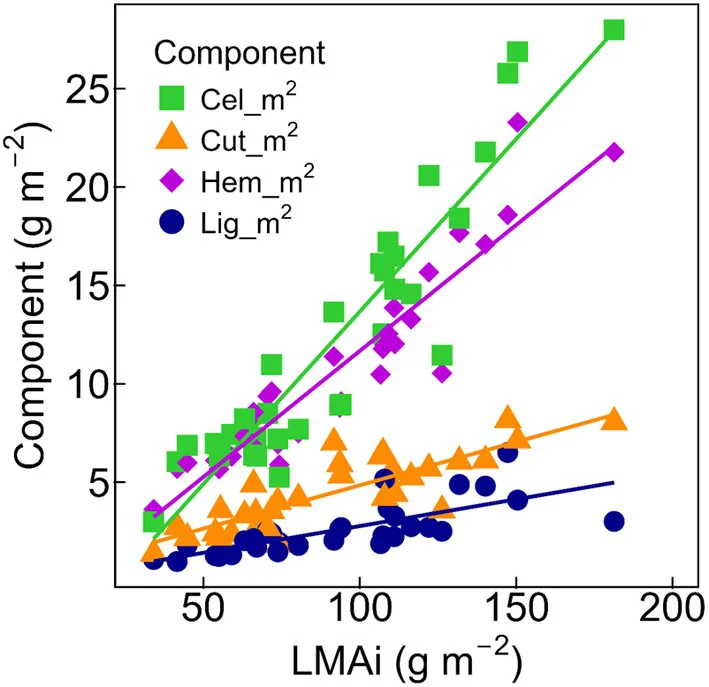
Correlation between interveinal leaf mass per area (LMA_i_) and the fiber fraction on a leaf area basis (grams of each component per square meter). The four correlations are highly significant (*P* < 0.001). Slopes were: cellulose (Cel) = 0.175, hemicellulose (Hem) = 0.128, cutin (Cut) = 0.044, and lignin (Lig) = 0.027. Each point represents the average value for one species. LMA_i_ was calculated in eight leaves sampled from five individuals for each species (*n* = 33). Chemical composition was determined from one composite interveinal sample per species, analyzed in triplicate (technical replicates), and the mean value was used in all statistical analyses.

### Composition on a mass basis

After comparing the concentration of cell wall components in the fiber fraction between the two leaf habits (see Material and Methods for further details on the calculation and expression of these values), evergreen species contain significantly more cellulose than deciduous ones (Table 1). Nonetheless, deciduous species allocate significantly more hemicellulose and cutin per 100 g of fiber than evergreen ones (Figure 5a, Table 1). No significant differences are present in the content of lignin between deciduous and evergreen species (Table 1). The correlation between the different cell wall components and LMA_i_ shows no significant relationship except for cellulose (*R* ^2^ = 0. 322, *P* < 0.001) (Figure S6).

**Figure 5:**
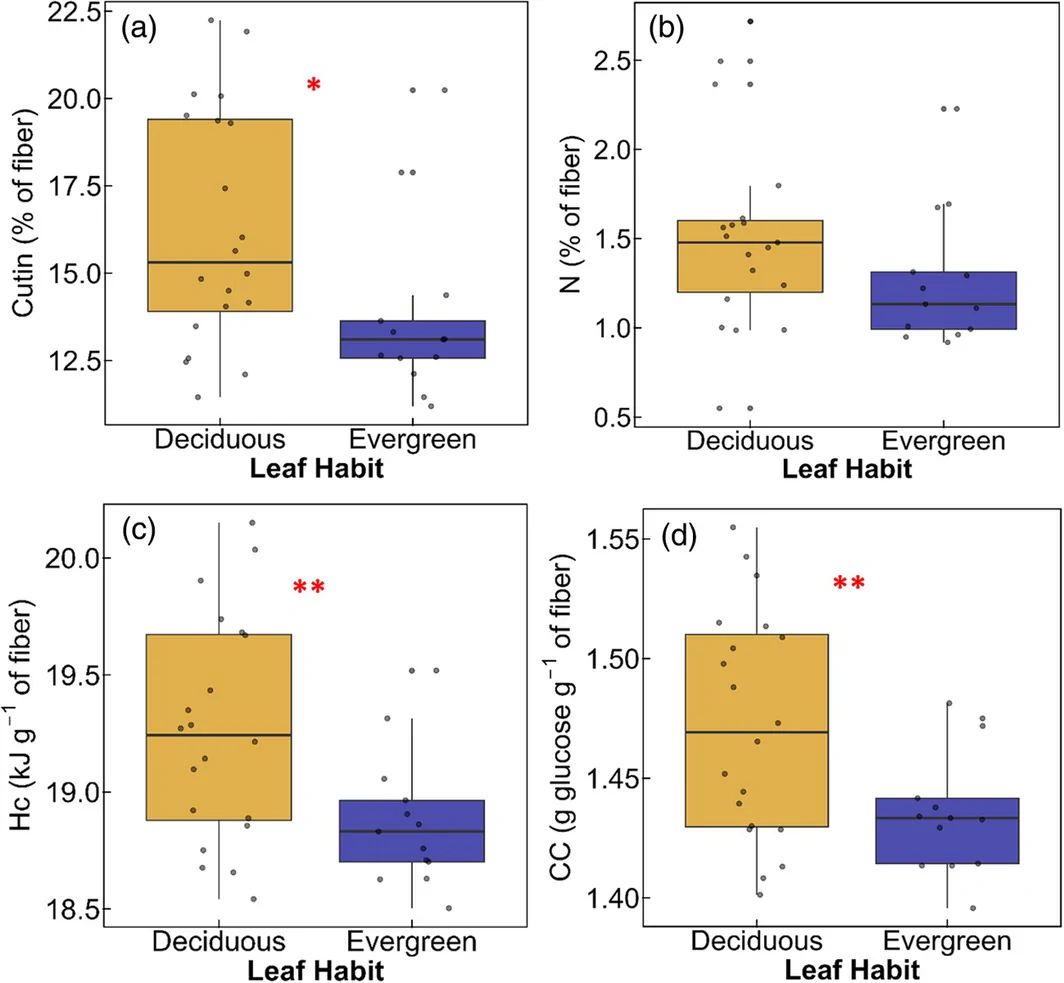
Boxplots of fiber‐fraction‐based traits comparing deciduous (orange, *n* = 20) and evergreen (blue, *n* = 13) species: (a) cutin concentration (% of fiber), (b) nitrogen concentration (% of fiber), (c) heat of combustion of the fiber fraction (kJ g^−1^ of fiber), and (d) construction cost of the fiber fraction (g glucose g^−1^ of fiber). Chemical composition was determined from one composite interveinal sample per species, analyzed in triplicate (technical replicates), and the mean value was used in all statistical analyses. Red asterisks indicate statistical significance levels based on *t*‐test/Wilcoxon tests: *P* < 0.05 (*), *P* < 0.01 (**), *P* < 0.001 (***), *P* > 0.05 (no asterisk).

The total nitrogen content per gram of dry mass is significantly higher in deciduous species than in evergreen ones (Table 1). However, no significant differences are present in the nitrogen content of the fiber fraction between both leaf habits (Figure 5b, Table 1).

Deciduous species release a significantly higher heat of combustion in the fiber fraction (Hc_fib) than evergreen species (Figure 5c, Table 1). Similarly, deciduous species show a significantly higher construction cost to produce their cell walls than evergreen ones (Figure 5d, Table 1).

### Functional relationships between construction costs and mechanical properties

A weak but significant negative correlation is present between *F*_max_ and the construction cost of the fibers (*R* ^2^ = 0.186, *P* = 0.012) (Figure S7). Deciduous species have, on average, low values of *F*_max_, but a wide range of variation in the construction cost of the fibers (CC_fib). Evergreen species tend to exhibit low values of CC_fib, but a wider range of variation in the *F*_max_. As expected, the construction cost of the fibers is significantly correlated with both of the variables used to calculate it, namely, the heat of combustion of the fiber fraction (*R* ^2^ = 0.872, *P* < 0.001; Figure 6a) and nitrogen content in the fiber fraction (*R* ^2^ = 0.434, *P* < 0.001; Figure 6b). Besides, a significant positive correlation is also present between the CC_fib and the cutin concentration in the fiber fraction (*R* ^2^ = 0.571, *P* < 0.001; Figure 6c). However, there is a significant negative correlation between CC_fib and the cellulose concentration in the fiber fraction (*R* ^2^ = 0.379, *P* < 0.001; Figure 6d). Deciduous species with the highest values of CC_fib show high values of nitrogen concentration in the fiber fraction (N_fib), heat of combustion of the fiber fraction (Hc_fib), and cutin concentration in the fiber fraction (Cut_fib) (Figure 6(a–c)). On the other hand, evergreen species with the highest values of *F*_max_ show the highest concentrations of cellulose in their fibers (Figure 6d). A highly significant relationship was evident between construction cost per unit area and LMA_i_ (*R* ^2^ = 0.885, *P* < 0.001), with a slope of 0.51 (data not shown).

**Figure 6:**
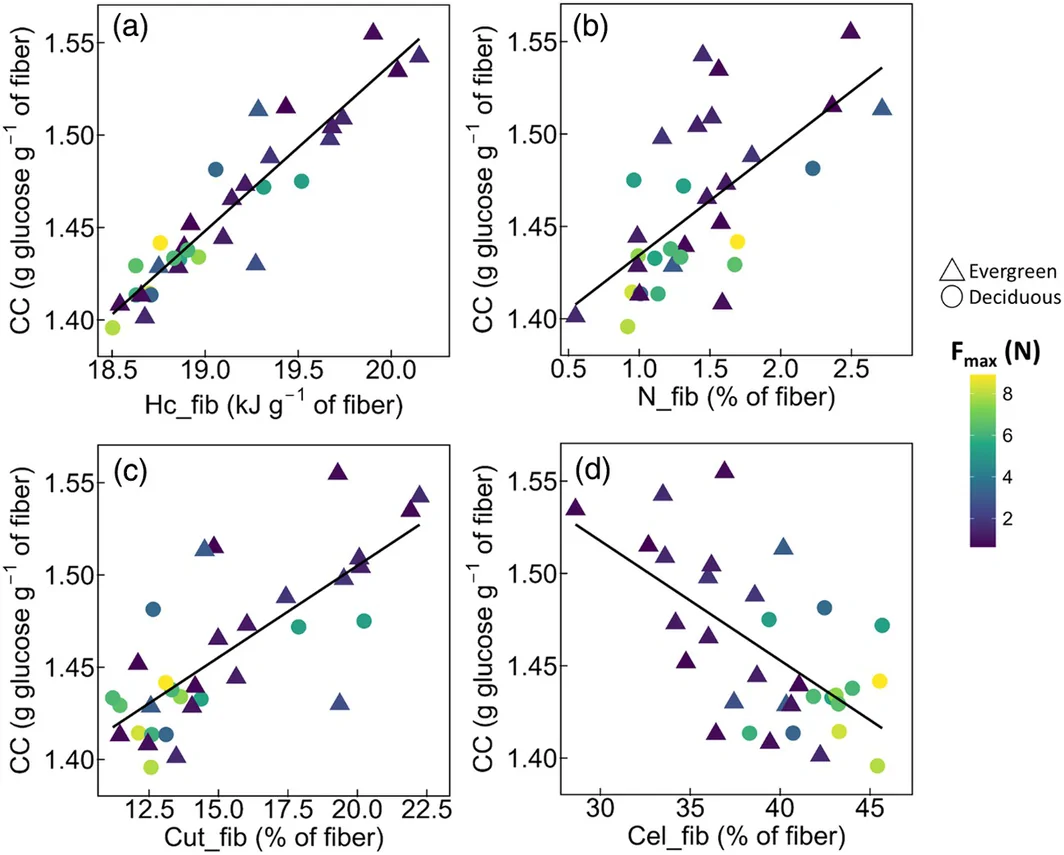
Correlation between construction cost (CC) of the fiber fraction and: (a) Heat of combustion of the fiber fraction (Hc_fib), (b) Nitrogen of the fiber fraction (N_fib), (c) Cutin of the fiber fraction (Cut_fib), and (d) Cellulose of the fiber fraction (Cel_fib). Each point is the average value for a species. Triangles represent evergreen species (*n* = 13) and circles represent deciduous ones (*n* = 20). Chemical composition was determined from one composite interveinal sample per species, analyzed in triplicate (technical replicates), and the mean value was used in all statistical analyses. Maximum force (*F*_max_) is represented as a third variable encoded by a color gradient.

## DISCUSSION

### Morphological and anatomical determinants of mechanical reinforcement in evergreen and deciduous oaks

The interveinal LMA (LMA_i_) showed clear divergence between leaf habits, with evergreen species generally exhibiting higher values of LMA_i_ than deciduous ones, as reported in other studies (Sancho‐Knapik et al., 2021). LMA_i_ values reported in this study overlapped substantially between deciduous and evergreen species. Within this overlapping range, leaves with similar LMA_i_ can vary widely in lifespans—from tropical evergreens whose leaves may persist for more than 3 years (e.g., *Quercus myrsinifolia* Blume) to Mediterranean deciduous oaks with lifespans of around 8 months (e.g., *Q. faginea* Lam.) (Harayama et al., 2016; Mediavilla & Escudero, 2009). Although LMAi remained strongly correlated with *F*_max_, consistent with previous work (Alonso‐Forn et al., 2023; Onoda et al., 2011), the overlap in LMA_i_ contrasted with the almost complete separation in *F*_max_ between leaf habits. Moreover, the relationship between LMA_i_ and *F*_max_ differed significantly between deciduous and evergreen species, with evergreen species exhibiting a steeper increase in mechanical strength with increasing LMA_i_. Evergreen species consistently achieved higher *F*_max_ than deciduous species at comparable LMA_i_ values. These results indicate that similar structural investment per unit area does not necessarily translate into similar mechanical properties across leaf habits. A first explanation for this pattern could be the generally greater lamina thickness of evergreen leaves. Indeed, thickness was also significantly correlated with *F*_max_. Although deciduous species do not reach the high thickness values of evergreens, their lamina thickness increases with increasing LMA_i_. Besides directly enhancing mechanical properties, greater thickness may also provide functional benefits for photosynthesis. Increased thickness can be associated with greater mesophyll and chloroplast surface area available for CO_2_ diffusion, potentially enhancing mesophyll conductance and photosynthetic capacity (Mizokami et al., 2022). However, this relationship is not necessarily straightforward, as increased thickness may also involve greater cell wall investment, which increases construction costs while potentially constraining CO_2_ diffusion. Thus, the photosynthetic consequences of greater LT likely depend on the balance between these structural costs and benefits, as well as on environmental conditions and leaf longevity (Mizokami et al., 2022). In this context, the greater mesophyll nitrogen per unit area and consequently greater Rubisco content associated with thicker leaves may represent an additional mechanism contributing to their higher photosynthetic capacity (Peguero‐Pina, Sisó, et al., 2017). Besides, greater LT may also reduce leaf water loss (Lamont et al., 2025).

At first glance, this could indicate that thickness alone drives the higher *F*_max_ in evergreens. However, after accounting for the effect of thickness by analyzing specific strength (SS), the significant relationship between SS and LMA_i_ remained; such differences in lamina thickness alone cannot explain the greater mechanical properties of evergreen species at comparable LMA_i_. Together with the steeper *F*_max_–LMA_i_ relationship observed in evergreen species, these results show that a given increase in structural investment yields a larger gain in mechanical properties than in deciduous species. Such enhanced resistance likely reflects differences in internal anatomical investment. Potential contributors include a higher proportion of small xylem vessels (Alonso‐Forn et al., 2023; Martín‐Sánchez et al., 2025), greater amounts of sclerenchyma and collenchyma in bundle sheath extensions, thicker epidermal cell walls, and more cellular layers in the mesophyll (Alonso‐Forn et al., 2023; John et al., 2017; Kitajima et al., 2012; Mediavilla et al., 2008; Sancho‐Knapik et al., 2021). Indeed, Sancho‐Knapik et al. (2021) reported that increasing lamina thickness was associated with wider bundle sheath extensions, which were on average 1.3‐fold broader in evergreens than in deciduous species. This pattern implies that thicker leaves may also exhibit greater mesophyll reinforcement, helping prevent thickness shrinkage, especially in the case of evergreen oaks (Peguero‐Pina et al., 2023). Similarly, thickened cell walls increase the Young's modulus and ultimately contribute to resistance against deformation (Alonso‐Forn et al., 2020; Read & Sanson, 2003).

These structural reinforcements might be expected to increase tissue compactness. However, when leaf density—expressed as the ratio of LMA_i_ to lamina thickness—is considered, deciduous species are, on average, denser than evergreens, although an overlap exists between the densest deciduous and the least dense evergreen species. This pattern was also reported by Sancho‐Knapik et al. (2021), who suggested that differences in LT may contribute substantially to variation in leaf density when density is calculated as LMA divided by thickness. Therefore, the higher leaf density of deciduous species should not be interpreted as evidence of greater tissue reinforcement. Rather, higher bulk leaf density does not necessarily imply greater structural reinforcement, because this metric integrates several structural components and does not capture their anatomical arrangement. To better understand internal structural reinforcement, it is more informative to consider tissue densification rather than bulk leaf density. A study by John et al. (2017) exemplifies this approach, showing through a mathematical decomposition of LMA that the two most relevant predictors are the number of cell layers and the tissue dry mass density, defined as the amount of dry matter (cell wall plus protoplast) per unit volume of the non‐air space of mesophyll and epidermal cells. In this framework, leaves achieve greater “densification” through smaller mesophyll and epidermal cells and/or a higher number of cell layers—anatomical configurations that characterize evergreen species and, correspondingly, those with higher values of LMA.

Already in 1986, two complementary perspectives highlighted the adaptive significance of such designs. Grubb (1986) distinguished two models of sclerophyllous: pachyphylls, with thick mesophyll but low‐density spongy parenchyma, and pycnophylls, with compact mesophyll and reduced intercellular spaces. Within *Quercus*, true pachyphylls are absent, but several Mediterranean evergreen oaks conform to the pycnophyll type, particularly those evergreens with LMA_i_ values above the median for this leaf habit. Oertli (1986) showed that smaller cells can better withstand collapse during dehydration. This idea was later supported by Ding et al. (2014), who added that, beside smaller cell sizes, thicker walls also help to mitigate excessive dehydration by buffering against negative turgor pressures. Moreover, cell size may have broader functional consequences, as smaller cells can increase the surface area of chloroplasts exposed to intercellular air spaces, thereby facilitating CO_2_ diffusion, while also contributing to greater mechanical strength (Terashima et al., 2001). Beyond conferring advantages under drought, a higher LMA may also enhance tolerance to cold stress. Among evergreen species, those from Mediterranean environments face a particularly demanding seasonal stress regime, with both intense summer drought and low winter temperatures (Martín‐Sánchez et al., 2022). This combination of hydraulic and thermal constraints likely imposes strong selective pressure favoring structurally reinforced leaves with high LMA, capable of maintaining integrity and functionality across contrasting stress conditions. Indeed, González‐Zurdo et al. (2016) showed that in species experiencing cold winters, LMA increases primarily through greater lamina driven by the accumulation of structural carbohydrates (cellulose and hemicellulose) in the cell walls, rather than through changes in tissue density or lignin content. Such thicker, reinforced leaves are better able to withstand freezing and slow internal ice propagation, which contributes to longer LLS and allows perennials to amortize their construction costs.

### Chemical composition of the interveinal fiber fraction

Having established that evergreen species exhibit higher LMA, thickness, and toughness—generally supported by greater structural reinforcement—the next step was to assess whether the chemical composition of their cell walls (i.e., the so‐called fiber fraction) reflects these physical properties, and whether enhanced mechanical resistance necessarily entails higher energetic investment.

When expressed per unit area, the abundance of all four polymers increased with LMA_i_, as expected because they constitute part of leaf mass. However, the unequal scaling slopes indicate that their relative contributions to increasing LMA_i_ differ. Cellulose and hemicellulose accounted for a proportionally larger share of the additional mass accumulated with increasing LMA_i_ than lignin and cutin. Importantly, this does not imply that evergreen species invest less in lignin or cutin. On the contrary, both compounds also increased with LMA_i_, likely reflecting thicker cuticles and, in some evergreen species, lignified epidermal tissues. Rather, cellulose and hemicellulose accumulated disproportionately faster, progressively diluting the contribution of the more metabolically expensive compounds to the overall fiber fraction.

The greater allocation of cutin in evergreen species is nevertheless of particular interest because cutin is concentrated in the cuticle, a surface structure whose investment is more directly related to leaf area than to leaf volume. Evergreen species had approximately 70% more cutin per unit leaf area than deciduous species, consistent with greater investment in cuticular material and, potentially, thicker cuticles. This difference may contribute to their greater mechanical resistance, as cuticle thickness has been shown to be positively related to leaf mechanical resistance (Onoda et al., 2012). Consistent with this interpretation, cutin concentration per unit area remained positively associated with mechanical properties even after accounting for LT through SS, indicating that its association with mechanical resistance is not simply a consequence of evergreen species producing thicker leaves. Although we did not directly quantify cuticle thickness or its spatial contribution to mechanical resistance, these findings are consistent with the possibility that cuticular reinforcement represents an additional component of the mechanical strategy of evergreen leaves, alongside their greater investment in structural fiber tissue. These compositional patterns naturally lead to the question of whether differences in fiber chemistry translate into differences in the energetic cost of constructing the structural fraction.

### Construction costs and the economics of structural investment

The compositional differences described above allowed us to evaluate whether evergreen species achieve greater mechanical reinforcement by constructing more expensive fibers or simply by producing larger amounts of structural tissue. Contrary to expectation, evergreen oaks did not possess more expensive fibers. Instead, deciduous species showed higher construction costs per unit fiber mass despite having mechanically weaker leaves. This apparent paradox is explained by differences in fiber composition. Evergreen species accumulate much larger amounts of cellulose and hemicellulose as LMA_i_ increases. Because these structural carbohydrates are metabolically less expensive than lignin and cutin, their massive accumulation effectively lowers the average construction cost of the fiber fraction on a mass basis. Conversely, the higher mass‐based fiber costs of deciduous species arise because the more expensive compounds are proportionally less diluted within a smaller structural framework. Thus, evergreen species do not achieve greater toughness by constructing more expensive fibers, but by producing substantially more fiber per unit leaf area while relying predominantly on relatively inexpensive structural carbohydrates. In other words, evergreen species improve mechanical properties primarily by increasing the amount of relatively inexpensive structural carbohydrates, rather than by increasing the energetic cost of the fiber itself. This compositional strategy is also functionally advantageous, as cellulose‐rich cell walls simultaneously enhance mechanical reinforcement and reduce tissue palatability to herbivores (Kitajima et al., 2012), providing an efficient means of increasing leaf persistence without proportionally increasing metabolic costs.

It is therefore important to distinguish between the construction cost of the fiber itself and the total construction cost of the leaf. Evergreen fibers are less expensive on a mass basis, but evergreen leaves contain substantially more structural material per unit area owing to their higher LMA. Consequently, evergreen species invest more resources overall in wall polymers and exhibit consistently higher area‐based construction costs than deciduous species, fully consistent with the predictions of the LES (Wright et al., 2004). Our analysis therefore refines previous observations that evergreen species exhibit higher whole‐leaf construction costs (Villar & Merino, 2001) by demonstrating that this reflects greater quantities of structural tissue rather than intrinsically more expensive fibers. Importantly, although construction cost depends on both heat of combustion and structural nitrogen, the higher mass‐based cost of deciduous fibers was explained almost entirely by their elevated heat of combustion, whereas wall‐bound nitrogen did not differ significantly between leaf habits.

It is important to note, however, that estimates of wall‐bound nitrogen obtained through detergent‐based extraction entail inherent methodological constraints. Heat and drying during sample preparation may cause a fraction of cytosolic proteins to become insoluble or bind to phenolic compounds, potentially remaining in the residue and leading to a slight overestimation of cell wall nitrogen (Onoda et al., 2017). Conversely, some wall‐associated proteins are only weakly bound to the polysaccharide matrix and can be solubilized and lost during extraction when relatively strong solvents are used, resulting in a potential underestimation of genuine wall nitrogen (Fry, 1988; Lamport, 1966; Onoda et al., 2017). These opposing biases cannot be fully disentangled with the present approach and should therefore be considered when interpreting absolute values. Nevertheless, because the same protocol was applied consistently across all species, the absence of systematic differences in wall‐bound nitrogen between deciduous and evergreen oaks remains robust at the comparative level.

The lower mass‐based construction costs of evergreen fibers also have implications for interpreting payback time within the LES, although this concept should not be confused with whole‐leaf construction costs. Payback depends on the total construction cost per unit leaf area relative to lifetime carbon gain, rather than on the cost of individual fiber components (Poorter et al., 2006; Ye et al., 2022). Accordingly, although evergreen fibers are cheaper to construct per unit mass, evergreen leaves remain more expensive overall because they contain substantially more structural biomass per unit area. The characteristically longer payback times of evergreen species (Eamus & Prichard, 1998; Sobrado, 1991) therefore most likely reflect ecological rather than compositional constraints. For example, in Mediterranean environments, where both summer drought and winter low temperatures restrict the period during which leaves can sustain high carbon gain (Corcuera et al., 2004; Limousin et al., 2010; Mediavilla & Escudero, 2003b), the prolonged lifespan of evergreen leaves allows these greater construction costs to be amortized over several years (Harayama et al., 2016; Mediavilla & Escudero, 2003a; Niinemets, 2015). Still, deciduous species also occur in Mediterranean climates, often co‐existing with evergreens under the same stresses (Gil‐Pelegrín et al., 2017). These species typically invest more in fiber construction yet maintain a much shorter LLS, lasting only a few months. Their instantaneous photosynthetic rates are not substantially higher than those of evergreen species, but they adopt a less conservative strategy, maximizing carbon gain within a narrow time window (Peguero‐Pina, Aranda, et al., 2017). Indeed, Mediterranean deciduous species are the only deciduous taxa in our dataset that can be considered true sclerophylls, displaying anatomical similarities to Mediterranean evergreens. Although not true pycnophylls, their enhanced sclerophylly—which ultimately reflects higher construction costs—may be an adaptation to withstand Mediterranean stress.

## CONCLUSION

Evergreen oaks on average displayed 70% higher LMA_i_ values than deciduous species, yet leaf habit alone did not fully define this trait, as some deciduous species regarded as sclerophylls and certain evergreens remain orthophylls. Importantly, while LMA_i_ values overlap between habits, this is not the case for mechanical properties: evergreen oaks consistently exhibited higher maximum force than any deciduous species considered in this study, even at similar levels of LMA_i_. This indicates that their higher toughness derives not merely from greater biomass investment on an area basis, but from specific anatomical reinforcements built predominantly from metabolically inexpensive polymers such as cellulose and hemicellulose. A key and novel result is that, on a dry mass basis, deciduous species produce more expensive fibers, whereas evergreen toughness arises from the amount of tissue invested rather than its intrinsic chemical cost. Methodologically, our study advances this field by quantifying lignin and cutin separately—a distinction rarely made in leaf economics research—and by showing that our construction cost estimates, derived without direct calorimetry, correlate strongly with combustion‐based measures.

## MATERIALS AND METHODS

### Plant material

For this study, we sampled leaves of 33 oak species from our living collection in CITA de Aragón (41°39′ N, 0°52′ W, 200 m a.s.l., Zaragoza, Spain). Individual trees show some variability in age and size; however, all specimens were well‐established and grown under uniform Mediterranean outdoor conditions (mean annual temperature 15.4 °C, total annual precipitation 298 mm), with drip irrigation every 2 days. Tree age is approximately 5–10 years, and height ranges from approximately 30 cm to approximately 1.5 m depending on species and growth rate. Between 20 and 60 (depending on leaf size) current year fully developed mature leaves were collected in July from south‐exposed branches of five individuals per species during the early morning. Leaves were stored in plastic bags and immediately transported to the laboratory for mechanical testing and sample preparation.

### Punch‐and‐die test

Punch‐and‐die tests consist of a flat‐ended cylinder (punch) that moves at a constant speed against a leaf which is attached to a flat surface with a hole (die) below it. The force needed to punch the leaf is recorded at regular time intervals with a sensor. We followed the procedure used by Alonso‐Forn et al. (2023) using the same device: the force sensor (S2M/50 N Force Transducer; HBM, Germany) was attached to the moving head of the mechanical tester (Mach‐1 V500C MA001 system; Biomomentum, Québec, QC, Canada). A flat‐ended cylindrical punch made of steel of 2 mm diameter, with a clearance of 0.05 mm was mounted onto the force sensor. The die made of steel of 2.1 mm diameter was fixed in the base of the machine. The movement speed was fixed to 30 mm min^−1^ for all trials and force measurements were recorded at a rate of 100 Hz to generate force–displacement curves (Figure S1). A blank test was performed between measurement tests as a calibration to minimize the load forces related to the background friction caused by the proximity of the die walls to the punch tip. One test was carried out per leaf on eight leaves per species avoiding punching the midrib and secondary veins (Figure S1). This region was selected to standardize measurements across leaves and to avoid major veins, which have substantially higher structural reinforcement and would artificially increase force values while not being representative of the lamina tissue. Leaves were selected from five individuals per species, with a balanced but not identical contribution per individual, in order to minimize within‐individual pseudoreplication. All the mechanical tests were performed at room temperature with leaves fully hydrated.

LT was measured with a micrometer (GT‐H10L; Keyence, Osaka, Japan) attached to an amplifier unit (GT‐75AP; Keyence) immediately before each punch‐and‐die test.

Five mechanical traits were obtained by analyzing the recorded curves: maximum force (*F*_max_), defined as the highest load value; punch strength (S), calculated as *F*_max_ divided by the circumference of the punch, thereby standardizing force values for punch size and facilitating comparisons among studies using different punch diameters; work of fracture (WF), calculated as the area below the curve between the initial contact of the punch with the leaf and *F*_max_; specific punch strength (SS), calculated as S divided by LT; and specific work of fracture (SWF), calculated as WF divided by LT. We set the starting point of each curve from 10% of *F*_max_ to avoid the effect of the three‐dimensional structure of the leaf (Alonso‐Forn et al., 2023). See all traits, abbreviations, and units in Table 2. Pairwise correlation analyses among the five mechanical traits revealed strong correlations (Figure S2); therefore, we used *F*_max_ as the main representative of leaf mechanical properties, given that it is the most straightforward variable. Specific punch strength (SS) was additionally used where a thickness‐independent measure of mechanical properties was required.

**Table 2:** List of every trait measured, meaning of the abbreviations used and units

Trait	Definition	Unit
LA	Leaf area	cm^2^
LMA_l_	Leaf mass per area of the whole leaf	g m^−2^
LMA_i_	Interveinal leaf mass per area	g m^−2^
LT	Leaf thickness	mm
Density	Leaf density	g cm^−3^
*F*_max_	Maximum force	*N*
S	Punch strength	kN m^−1^
SS	Specific punch strength	MN m^−2^
WF	Work of fracture	J m^−1^
SWF	Specific work of fracture	kJ m^−2^
Cell content	Percentage of cellular content	%
Fiber fraction	Percentage of fiber	%
Fib_m^2^	Total fiber content per square meter	g m^−2^
Cel_m^2^	Cellulose concentration per square meter	g m^−2^
Hem_m^2^	Hemicellulose concentration per square meter	g m^−2^
Lig_m^2^	Lignin concentration per square meter	g m^−2^
Cut_m^2^	Cutin concentration per square meter	g m^−2^
N_m^2^	Nitrogen concentration per square meter	g m^−2^
Hc_m^2^	Heat of combustion per square meter	kJ m^−2^
CC_m^2^	Construction cost per square meter	g glucose m^−2^
Cel_fib	Cellulose concentration in the fiber fraction	% of fiber
Hem_fib	Hemicellulose concentration in the fiber fraction	% of fiber
Lig_fib	Lignin concentration in the fiber fraction	% of fiber
Cut_fib	Cutin concentration in the fiber fraction	% of fiber
N_fib	Nitrogen concentration in the fiber fraction	% of fiber
N_Total	Total nitrogen concentration in the whole interveinal area	% of dry mass
Hc_fib	Heat of combustion of the fiber fraction	kJ g^−1^of fiber
CC_fib	Construction cost of the fiber fraction	g glucose g^−1^ of fiber

### Morphological traits and compositional content

In this study, we have targeted the interveinal lamina since punch‐and‐die tests are performed in this area of the leaf. Here, we use “interveinal” to refer operationally to the lamina region between secondary veins, excluding the primary and secondary veins themselves. Although minor tertiary veins may remain within these sections, they were not removed, as their manual separation is impractical and could introduce substantial sampling variability. This approach was adopted to consistently exclude the major vascular structures while retaining the lamina in which mechanical measurements are performed. This region represents the photosynthetically active portion of the leaf, excludes the major vascular structures, and is most relevant for estimating both mechanical properties and chemical investment. Thus, the subsequent measurements—LMA and fiber content—were taken in such areas. The leaf area between two secondary veins was cut and both area and dry mass were measured to get the LMA_i_ in eight leaves per species. The leaf area was measured using the software ImageJ. Dry mass was obtained by weighing after 3 days of oven‐drying at 70°C. Then LMA_i_ was calculated as the ratio between the interveinal dry mass and the interveinal leaf‐section area. Additionally, we calculated the LMA of the whole leaf (LMA_l_) in eight leaves per species, by scanning such leaves and measuring both their leaf area and dry mass. The LMA_l_ was calculated as the ratio between the dry mass and the whole leaf area. Finally, the leaf density was calculated as LMA_i_ divided by LT (Sancho‐Knapik et al., 2021).

The next step was to quantify the fiber content (i.e., the cell wall fraction) as well as the different components in such cell walls: cellulose, hemicellulose, lignin, cutin, and proteins (nitrogen content). To ensure consistency with mechanical tests, the plant material consisted exclusively of interveinal lamina, collected in enough quantity to obtain at least 3 g of dry mass per species. Thus, during the sample preparation, we removed the petiole, midrib, and secondary veins, since they are mainly vascular tissue rich in xylem and sclerenchyma and therefore, in lignin. We also removed the peripheral tissue of leaf laminae (Figure S3). The resulting material was then oven‐dried 3 days at 70°C, ground and stored in a desiccator. Then, we followed a gravimetrical procedure based on the method proposed by Goering and Van Soest (1970) and Van Soest et al. (1991) modified by Mertens (2002) using the Ankom 200/220 fiber analyzer (Ankom Technology Corporation, Fairport, NY, USA) with two additional modifications by ourselves (Figure 7). First, fiber filter bags were used instead of crucibles. Besides, we added two additional steps to the original procedure of Van Soest et al. (1991): (i) an AIR extraction series (Fangel et al., 2021; Nadal et al., 2020), and (ii) lignin extraction throughout NaClO_2_ (Espinosa, 2019; Jin et al., 2019; Morcillo‐Martín et al., 2022). For each species, interveinal tissue collected from five individuals was pooled into a single composite sample. Three independent analytical replicates were performed on subsamples of each composite sample for the Van Soest fiber analyses, and their mean value was used for subsequent statistical analyses.

**Figure 7:**
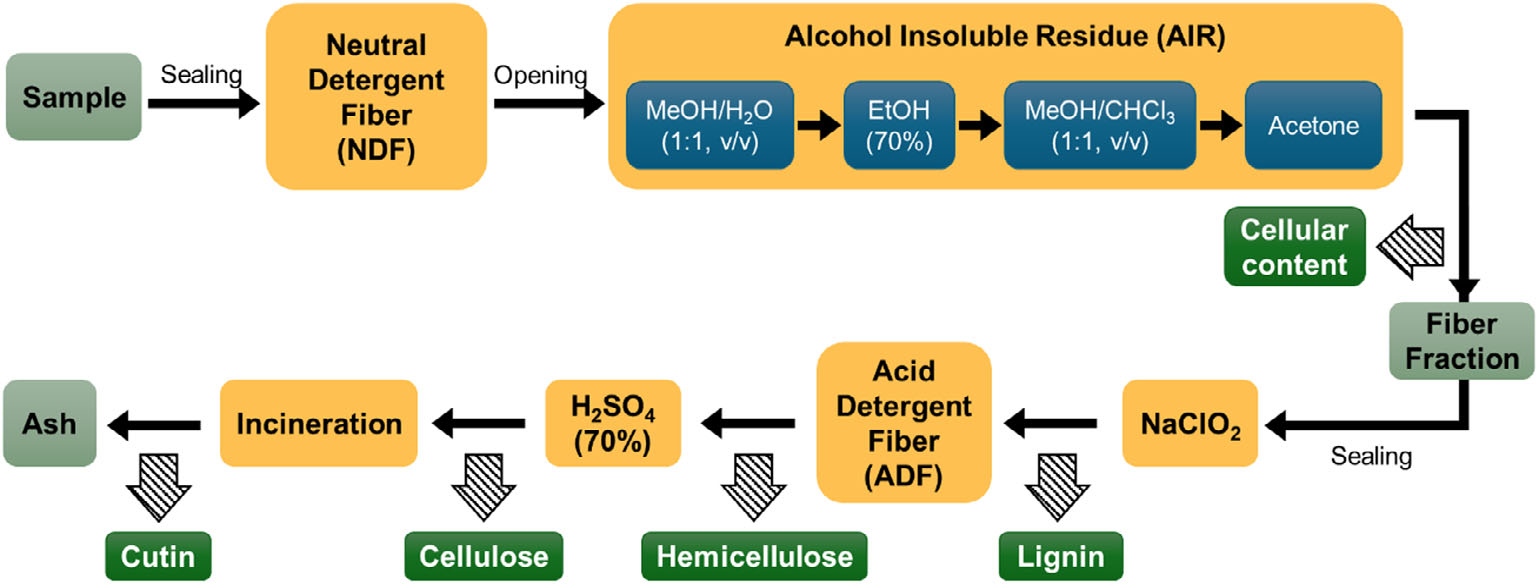
Schematic representation of the protocol used for the fractionation and quantification of structural components. Orange squares represent each of the chemical procedures used in each step and hatched arrows show the component removed after each procedure.

The complete procedure was as follows. First, fiber filter bags (25 μm porosity, Ankom Technology Corporation, Fairport, NY, USA) stored in a desiccator were weighed and used to seal 0.5 g of ground material. Then, series of 48 bags were submerged in 2 L of NDF plus 20 g of Na_2_SO_3_ and 4 ml of α‐amylase solution and heated to 100°C (Van Soest et al., 1991) (Figure 7). After an hour, bags were rinsed in hot water and the cycle was repeated four times. Finally, bags were cleaned with acetone, dried and weighed to calculate the theoretical fiber fraction according to the original protocol. However, most of our samples still showed greenish colors in the acetone cleaning, indicating the presence of cellular content in the samples. For that reason, we decided to add AIR extractions after the NDF to ensure a more reliable value of the fiber and cellular content (Fangel et al., 2021; Nadal et al., 2020). Bags were opened and the material was homogenized for the same species. This material was treated in an organic solvent series: MeOH/H_2_O (1:1, v/v), EtOH (70%), MeOH/CHCl_3_ (1:1, v/v) and acetone, respectively (Figure 7). Each extraction consisted of adding 15 ml of solvent to the plant material, shaking for an hour to 1400 rpm, allowing the solid fraction sediment for 24 h, removing the supernatant, and repeating the same solvent twice before proceeding to the next solvent. After the last extraction with acetone, the resultant material is oven‐dried and weighed. The fiber fraction is calculated as the residual weight remaining after the NDF + AIR extractions, expressed as a proportion of the initial dry mass.

The first compound to quantify from the fiber fraction was lignin (Figure 7), since it forms lignocellulosic compounds (also known as recalcitrant cellulose) that can hinder the subsequent quantification of the cellulose (Espinosa, 2019; Hu & Ragauskas, 2012). The fiber fraction after the AIR was re‐sealed in new fiber filter bags again. A series of 12 bags were submerged in 500 ml of acetic acid (1%) and heated to 80°C. When the temperature was reached, 0.3 g of NaClO_2_ per sample gram was added and the bags were shaken during 1.5 h (Espinosa, 2019). The solution had to remain yellowish and the bags completely white; otherwise, the process was repeated. Then, the bags were cleaned with distilled water and acetone, oven‐dried for 24 h, and weighed. To ensure that we effectively removed all the lignin, we opened some testing bags, stained the samples with phloroglucinol solution (7.5 ml of EtOH 99.9%, 2.5 ml of HCl 37%, and 0.2 g of phloroglucinol), and observed them in the optical microscope (Pomar et al., 2002; Ruzin, 1999). The absence of reddish stained material implied the effective removal of the lignin.

The next steps followed the rest of the procedures of the original protocol of Van Soest et al. (1991). In short, bags were treated with ADF (acid detergent fiber) to remove hemicelluloses, then with sulfuric acid (72%, w/w) to remove the cellulose fraction. Again, we opened some testing bags and stained them with calcofluor‐white (0.5% w/v) and observed them in the optical microscope with ultraviolet light. The absence of bright blue colors indicates the absence of cellulosic traces. Finally, they were burned at 500°C in a muffle to quantify the ash fraction. The cutin fraction is assumed to be the weight after the cellulose removal minus the weight of the ash fraction (Figure 7).

Finally, nitrogen content was measured using an organic element analyzer (Flash EA 112; Thermo Fisher Scientific, Waltham, MA, USA). We calculated the total amount of nitrogen in the leaves using 100 mg of the initial ground material and we estimated the total amount of nitrogen in the cell walls using 100 mg of the material after the NDF + AIR process.

The different abbreviations and definitions of the fractions can be seen in Table 2. To express the amount of each chemical component per unit leaf area (g m^−2^), we multiplied their mass fractions (g g^−1^ dry mass) by LMA_i_ (leaf mass per area, g m^−2^). This conversion allows us to assess the absolute amount of each compound invested per square meter of leaf. In summary, since both LMA_i_ and fiber fraction are known for each species, the relative amount of each chemical compound can be expressed in two complementary ways: (i) relative to its proportion within the fiber fraction, or (ii) per unit leaf area.

### Heat of combustion and construction costs

To estimate the heat of combustion (*Hc*), we adopted a simplified, yet systematic, approach based on the chemical composition of leaf tissues. We calculated the *Hc* of the components representing the main structural fractions: cellulose, hemicellulose, lignin, cutin, and proteins. Cellulose, xylan (as hemicellulose), and lignin are sold as pure compounds for analytical purposes. Gluten was selected as the primary representative of plant‐derived protein. To determine the heat of combustion of cutin, we required a sufficient amount of isolated cutin material. Since obtaining the necessary quantity from oak leaves would have required extensive dissection of small leaf areas, we instead processed 1 kg of leaves from *Ligustrum vulgare*, a species with higher cutin content and larger, more easily dissected leaves. The tissue was subjected to the same sequential extraction steps used for oaks (NDF, AIR, ADF, lignin, and cellulose removal; see Figure 7) to isolate the cutin fraction. Importantly, we omitted the final incineration step, which in the analytical protocol is only used to determine ash content and would otherwise eliminate the cutin required for calorimetry.

The heat of combustion was determined in triplicate samples of 1.0–1.2 g for each chemical compound with an oxygen bomb calorimeter (1341 Plain Jacket Oxygen Bomb Calorimeter (Parr) No. 204 M, Parr Instrument Company, Moline, IL, USA). Subsequently, we derived species‐level heat of combustion values by computing the weighted arithmetic sum according to the relative abundance of each compound (assuming that essentially all nitrogen is allocated to protein) in the fiber fraction.

Finally, the leaf construction cost (CC) was calculated following the equation proposed by Williams et al. (1987):

$$\mathrm{CC}=\frac{\left(0.06968\mathrm{Hc}-0.065\right)\left(1-\left[A\right]\right)+\frac{7.5\mathrm{kN}}{14.0067}}{\mathrm{Eg}}$$

where *Hc* is the heat of combustion (in kilojoules per gram), [A] is the ash fraction (in grams of ash per gram of dry mass), *N* is the nitrogen concentration (in grams per gram of dry mass), *Eg* is the growth efficiency (0.89 for woody leaves; Williams et al., 1987), and *k* is the oxidation state of the nitrogen source (+5 for nitrate or −3 for ammonium), which is assumed to be +5 in well‐aerated Mediterranean soils (Alonso‐Forn et al., 2023).

While we acknowledge that this method does not fully capture the complex molecular interactions and bonding present in intact tissues—potentially influencing the actual heat of combustion—it offers a pragmatic and consistent alternative that enables cross‐species comparison and functional inference. Obtaining sufficient interveinal leaf material from all species for direct bomb calorimetry analysis would have represented a major methodological challenge, being both technically demanding and highly time‐consuming, and thus unfeasible within the scope of this study. To evaluate the reliability of the estimation approach, the actual construction cost of the fiber fraction was experimentally determined in five species (*Q. agrifolia* Née, *Q. faginea*, *Q. macrocarpa* Michx., *Q. phillyreoides* A. Gray, and *Q. rotundifolia* Lam.). The results showed a highly significant correlation between accurately measured and estimated values (*R* ^2^ = 0.979, *P* < 0.001), supporting the validity of the estimation method across species. This trade‐off was therefore considered justified in the context of the study's broader comparative framework.

### Statistical analyses

Statistical analyses were performed to evaluate differences between leaf habits (deciduous versus evergreen) and to assess functional relationships among structural, chemical, and mechanical traits. Prior to group comparisons, data were tested for normality and homogeneity of variances using Shapiro–Wilk and Levene tests, respectively. When assumptions of normality and homoscedasticity were met, differences between leaf habits were evaluated using Student's *t*‐tests; otherwise, the non‐parametric Wilcoxon rank‐sum test was applied. All hypothesis tests were conducted using two‐tailed tests, with statistical significance accepted at *P* < 0.05.

Relationships between continuous variables were analyzed using linear regression models. These analyses were used to examine the association of LMA_i_ with mechanical and compositional traits, as well as the relationships between fiber construction costs and their underlying chemical components. To assess whether the relationships between mechanical traits and leaf morphological traits differed between deciduous and evergreen species, we fitted linear models including leaf habit and its interaction with the explanatory variable (Response variable ~ Predictor × Leaf habit). The significance of the interaction term was used to test for differences in slope between leaf habits. For all regressions, the coefficient of determination (*R* ^2^) and associated *P*‐values are reported. Statistical significance was accepted at *P* < 0.05.

To further explore trait variation directly related to mechanical strength within each leaf habit, species were additionally classified into low‐ and high‐value groups for LMA_i_, LT, density, and *F*_max_ using the median value of each trait within each leaf habit as the threshold. This yielded four groups for each trait (deciduous‐low, deciduous‐high, evergreen‐low, and evergreen‐high). Differences among groups were evaluated using one‐way ANOVA followed by Tukey's HSD *post hoc* tests. This complementary analysis was used to compare the extreme portions of the trait distributions between leaf habits. Then, to quantify the degree of overlap between leaf habits, kernel density estimates were calculated for these same four traits. The overlap coefficient (OVL) was calculated as the shared area under the kernel density distributions, ranging from 0 (no overlap) to 1 (complete overlap). This analysis provided a quantitative measure of the extent to which the distributions of each trait were shared between deciduous and evergreen species. All analyses were performed in R (R Core Team, 2026).

#### Disclosure of AI Use

Artificial intelligence (AI) tools (Chat GPT, Open AI) were used to assist with language editing, improving the clarity and readability of the manuscript, refining sentence structure, and drafting text based on the authors' scientific content. AI was also used to assist in writing and debugging R code. All scientific decisions, study design, data analyses, interpretation of the results, and the final content of the manuscript were performed, verified, and approved by the authors, who take full responsibility for the accuracy and integrity of the work.

## Author Contributions

RM‐S and EG‐P conceived and designed the research; RM‐S performed the research and collected the data; RM‐S, DS‐K, JPF, and EG‐P analyzed the data. RM‐S wrote the first draft of the manuscript. All authors contributed to manuscript revision, approved the final version and agree to be accountable for all aspects of the work.

## Conflict of Interest

All the authors agree to declare no conflict of interest.

## Supporting information


**Figure S1.** Example of a force–displacement curve generated by the software during a punch test performed on a *Quercus variabilis* leaf. The punched area is indicated by a red dot. The purple dot shows the maximum force reached in this sample (0.901 N), and the vertical dashed line marks the time at which it occurred (4.82 s). The orange dashed line corresponds to 10% of the maximum force. Both dashed lines define the thresholds used to calculate the work of fracture (light‐blue shaded area).
**Figure S2**. Correlation matrix showing pairwise relationships among mechanical performance traits of leaves: maximum force (*F*_max_), strength (S), specific strength (SS), work of fracture (WF), and specific work of fracture (SWF). Values inside cells correspond to the significance level (*P*‐value), and background colors indicate the coefficient of determination (*R* ^2^) of each pairwise correlation. Eight leaves were sampled from five individuals per species (*n* = 33).
**Figure S3**. Example of the dissection procedure used to obtain the interveinal lamina sample for fiber analysis in *Quercus variabilis*. Starting from a fresh leaf (a), the petiole, midrib, secondary veins, and peripheral lamina tissue are removed (b). The resulting interveinal material (c) is then oven‐dried and ground prior to the fractionation and quantification of structural components (see Figure 7).
**Figure S4**. Kernel density distributions of LMA_i_ (a), leaf thickness (LT; b), leaf density (c) and maximum force (*F*_max_; d) for evergreen (dark blue, *n* = 13) and deciduous (orange, *n* = 20) species. The degree of overlap between leaf habits was quantified using the overlap coefficient (OVL): density = 0.567, LMA_i_ = 0.363, LT = 0.237 and *F*_max_ = 0.094. Eight leaves were sampled from five individuals per species (*n* = 33).
**Figure S5**. Correlation between interveinal leaf mass per area (LMA_i_) and specific strength (SS). Each point represents the mean ± SD of eight leaves sampled from five individuals for one species. Blue triangles represent evergreen species (*n* = 13) and orange circles deciduous ones (*n* = 20). Dashed colored lines represent the median value of each leaf habit for the LMA_i_. Linear regression line was fitted to the data. Since there was not a significant interaction (*P* = 0.788), a single regression line was fitted across species (*P* < 0.001).
**Figure S6**. Correlation between interveinal leaf mass per area (LMAi) and the fiber fraction on a fiber mass basis (% of fiber). The fitted line for cellulose (Cel) represents the only significant correlation (*P* < 0.001). Each point represents the average value for one species. LMA_i_ was calculated in eight leaves sampled from five individuals for each species (*n* = 33).
**Figure S7**. Correlation between maximum force (*F*_max_) and construction cost (CC) of the fiber fraction. Each point represents the mean of eight leaves sampled from five individuals for one species. Blue triangles represent evergreen species (*n* = 13) and orange circles deciduous ones (*n* = 20).

## Data Availability

The data that support the findings of this study are available in: https://doi.org/10.5281/zenodo.22705547.
