# Supplementary material for: Beyond leaf mass per area: anatomical and economic drivers of toughness in evergreen and deciduous oaks
Source: Plant J. 2026 Nov 11;128(3):e71217. doi: 10.1111/tpj.71217 (PMC13755502; doi:10.1111/tpj.71217)
Supplement: Supplementary file 1 [file TPJ-128-0-s001.docx]

**SUPPLEMENTARY DATA**

**Figure S1.** Example of a force–displacement curve generated by the software during a punch test performed on a Quercus variabilis leaf. The punched area is indicated by a red dot. The purple dot shows the maximum force reached in this sample (0.901 N), and the vertical dashed line marks the time at which it occurred (4.82 s). The orange dashed line corresponds to 10% of the maximum force. Both dashed lines define the thresholds used to calculate the work of fracture (light-blue shaded area).

**Figure S2.** Correlation matrix showing pairwise relationships among mechanical performance traits of leaves: maximum force (F_max_), strength (S), specific strength (SS), work of fracture (WF), and specific work of fracture (SWF). Values inside cells correspond to the significance level (p-value), and background colors indicate the coefficient of determination (R^2^) of each pairwise correlation. Eight leaves were sampled from five individuals per species (n = 33).

**Figure S3.** Example of the dissection procedure used to obtain the interveinal lamina sample for fiber analysis in *Quercus variabilis*. Starting from a fresh leaf (a), the petiole, midrib, secondary veins, and peripheral lamina tissue are removed (b). The resulting interveinal material (c) is then oven-dried and ground prior to the fractionation and quantification of structural components (see Fig. 1).

**Figure S4.** Kernel density distributions of LMA_i_ (a), leaf thickness (LT; b), leaf density (c) and maximum force (F_max_; d) for evergreen (dark blue, n = 13) and deciduous (orange, n = 20) species. The degree of overlap between leaf habits was quantified using the overlap coefficient (OVL): density = 0.567, LMA_i_ = 0.363, LT = 0.237 and F_max_ = 0.094. Eight leaves were sampled from five individuals per species (n = 33).

**Figure S5.** Correlation between interveinal leaf mass per area (LMA_i_) and specific strenght (SS). Each point represents the mean ± SD of eight leaves sampled from five individuals for one species. Blue triangles represent evergreen species (*n* = 13) and orange circles deciduous ones (*n* = 20). Dashed colored lines represent the median value of each leaf habit for the LMA_i_. Linear regression line was fitted to the data. Since there was not a significant interaction (*p* = 0.788), a single regression line was fitted across species (*p* < 0.001).

**Figure S6.** Correlation between interveinal leaf mass per area (LMAi) and the fiber fraction on a fiber mass basis (% of fiber). The fitted line for cellulose (Cel) represents the only significant correlation (p < 0.001). Each point represents the average value for one species. LMA_i_ was calculated in eight leaves sampled from five individuals for each species (n = 33).

**Figure S7.** Correlation between maximum force (F_max_) and construction cost (CC) of the fiber fraction. Each point represents the mean of eight leaves sampled from five individuals for one species. Blue triangles represent evergreen species (*n* = 13) and orange circles deciduous ones (*n* = 20).
